# Clustering of circumstances during the first 1000 days after conception and their association with school performance: a population-based cohort study from the Netherlands

**DOI:** 10.1136/bmjph-2024-002176

**Published:** 2025-08-17

**Authors:** Malon Van den Hof, Ilona Veer, Ruben van Gaalen, Tessa Roseboom

**Affiliations:** 1 Department of Epidemiology and Data Science, Amsterdam UMC, University of Amsterdam, Amsterdam, The Netherlands; 2 Amsterdam Reproduction and Development Research Institute, Amsterdam, The Netherlands; 3 Ageing & Later Life, Health Behaviours and Chronic Diseases, Amsterdam Public Health, Amsterdam, The Netherlands; 4 Statistics Netherlands, The Hague, The Netherlands; 5 Faculty of Social and Behavioural Sciences (Sociology), University of Amsterdam, Amsterdam, The Netherlands; 6 Department of Obstetrics and Gynaecology, Amsterdam UMC, University of Amsterdam, Amsterdam, The Netherlands

**Keywords:** Public Health, Education, Community Health, Humans

## Abstract

**Background:**

The first 1000 days of life are a crucial foundational period during which many different factors can impact development. It is unknown to what extent different factors cluster and how this affects later-life outcomes.

**Methods:**

In this population-based cohort study, we used registry data of all children born in the Netherlands in 2006. We used latent class analysis to investigate clustering of circumstances in the first 1000 days of life, including socioeconomic indicators (household income, parental education), prenatal and perinatal biomedical factors (maternal age, late-start antenatal care, preterm birth/born small for gestational age/poor start in life), and adverse childhood experiences in the first 1000 days (parental death, separation, mental health problems and detention) and associated clusters with school performance (ie, highest secondary school level advice at age 12).

**Results:**

In the study population of 181 575 children, we identified five clusters. We labelled cluster 1 (39%) and cluster 2 (27%) as ‘resource-richest’, clusters 3 (15%) and 4 (15%) as ‘intermediate’, and cluster 5 (5%) as ‘resource-poorest’, with the latter having the highest probabilities of low socioeconomic resources, adverse prenatal and perinatal biomedical factors and adverse childhood experiences in the first 1000 days. Compared with those in the resource-richest cluster (cluster 2), children in the resource-poorest cluster (cluster 5) had poorer school performance (OR 0.13, 95% CI 0.11 to 0.14), also after adjustment for parental education and household income (OR 0.20, 95% CI 0.18 to 0.24).

**Conclusions:**

Clustering of risk factors across different domains during the first 1000 days of life was associated with poorer school performance at age 12, suggesting that children growing up in resource-limited environments during this critical developmental window may face challenges in reaching their full developmental and educational potential. If we find similar associations with health-related outcomes, this would further underscore the importance of policies that strengthen resources across multiple domains early in life to support long-term human potential.

**WHAT IS ALREADY KNOWN ON THIS TOPIC**

- The first 1000 days of life represent a critical period for development and long-term health, influenced by a combination of socioeconomic factors, prenatal and perinatal biomedical conditions and adverse experiences.

**WHAT THIS STUDY ADDS**

- This study contributes to existing evidence by examining the clustering of various risk factors and resources during the first 1000 days of life in a comprehensive Dutch birth cohort.

**HOW THIS STUDY MIGHT AFFECT RESEARCH, PRACTICE OR POLICY**

- Our analysis revealed that limited resources across socioeconomic, prenatal and perinatal biomedical, and adverse experiences domains were strongly associated with poorer school performance at age 12, underscoring the multidimensional nature of early-life resources important in educational outcomes.

## Introduction

 The first 1000 days of a person’s life—the time spanning roughly from before conception to the child’s second birthday—is widely recognised as the most crucial developmental phase in which the foundations for development, growth and health are established. Many factors in this period impact human development and have potential irreversible effects.^1 2^

A body of evidence links circumstances during the first 1000 days of life to people’s ability to develop to their full potential. The environment in which humans develop from one single fertilised egg to a complete human being during the first 1000 days impacts brain development and thereby affects school performance and educational attainment.^2 3^ Socioeconomic circumstances during life, such as household income and parental education, are strongly related to children’s health and educational outcomes.^4^,^9^ Biomedical factors around pregnancy and birth also shape developmental potential. Children born preterm or small for gestational age (SGA) are more likely to experience cognitive and academic challenges compared with those born at term or with adequate birth weight.^10^,^13^ Beyond socioeconomic and biomedical influences, early-life experiences significantly impact a child’s development. Children who face adverse childhood experiences, such as violence, abuse, neglect, parental separation or loss or incarceration of a parent, not only have poorer health than adults but are also more likely to have chronic school absenteeism and poor education outcomes also after adjusting for sociodemographic variables.^14^,^18^ Taken together, these findings suggest that socioeconomic, biomedical and experiential factors during the first 1000 days of children’s development have a lasting impact on their ability to develop, with consequences for educational attainment and health. Although it is clear that these factors are important and that they are correlated, it is unclear to what extent these factors cluster in the Dutch population and what the relationship is between clustering of factors and later-life outcomes.

Previous research demonstrates that clustering of limited resources across multiple domains is associated with poorer development and lower educational achievement in later childhood.^19 20^ However, existing studies were relatively small and often did not capture all relevant aspects or domains as described above.

This study aimed to examine the clustering of socioeconomic, biomedical and experiential factors during the first 1000 days of life and their impact on later educational outcomes. Using population-based registry data of all children born in the Netherlands, we analysed how different risk factors in the first 1000 days of life cluster and assessed the association between these clusters and school performance at age 12.

## Materials and methods

### Data sources

We conducted the study in the Netherlands, a country with an area of 41 543 km^2^ and a population of 17.9 million people in 2023.^21 22^ This study used registry data from Statistics Netherlands, the Netherlands Perinatal Registry (Perined) and Vektis. The Statistics Netherlands serves as a Third Trusted Party for data linkage and covers integrated longitudinal micro data of numerous registers and surveys of the complete population in the Netherlands.^23^ The Perined registry is a national registry that contains data on pregnancy, obstetric history and pregnancy outcomes (eg, birth weight, gestational age, Apgar scores) from 22 weeks of gestation until 28 days after birth for more than 97% of all pregnancies in the Netherlands; these data are routinely collected by midwives, gynaecologists and paediatricians. Vektis provides data on healthcare utilisation and spending under the Dutch Healthcare Insurance Act and covers 99% of the Dutch (insured) population.^24^

### Study population

We included all children who were born in the Netherlands in 2006 and who had at least one legal parent registered with the Dutch Personal Records Database. We excluded children who died or migrated before the age of two because in those cases, we lacked registry data to examine available resources.

### Patient and public involvement

It was not appropriate or possible to involve patients or the public in the design, or conduct, or reporting, or dissemination plans of our research.

### Circumstances during the first 1000 days of life

To identify clusters of circumstances during the first 1000 days of life, we selected indicator variables using the following guiding principles: (1) variables relevant to health and development (supported by a theoretical framework and/or scientific evidence) and (2) variables relevant for the first 1000 days of life. We defined the first 1000 days of life as the period from conception to the second birthday. (3) Variables are available in data registries in the Netherlands. (4) The data are of good quality, as indicated by the data provider. (5) Variables have discriminative power in latent class analysis (LCA). (6) We selected variables to prevent too much overlap of variables essentially measuring the same construct. We recoded variables to obtain meaningful levels, with higher scores indicating the presence of risk. Using these guiding principles, we included nine indicator variables in three domains: (1) socioeconomic resources, (2) prenatal and perinatal biomedical factors and (3) adverse childhood experiences in the first 1000 days of life (table 1).

**Table 1:** Variables included in the latent class analysis

	Definition	Categories	Data source
Socioeconomic characteristics			
Household income	Standardised disposable income of private households in 2005 (year before birth). We categorised household income into the low (<40th percentile), moderate (40th–80th percentile) and high (>80th percentile). We averaged household incomes if parents did not share households.	Low, moderate, high	SSD
Parental educational level	The highest achieved education level of registered parents in 2005 (year before birth), categorised according to the International Standard Classification of Education (ISCED).^46^	Low, intermediate, high	SSD
Prenatal and perinatal biomedical factors			
Young age of the mother	Age of the mother at childbirth is below 21 years	No; yes	SSD
Late antenatal care initiation	Timing of first antenatal care visit, before or after 14 weeks of gestation.	No, yes	Perined
Preterm birth or born small for gestational age, or poor start in life	Preterm birth: born before 37 weeks of gestation. Small for gestational age: weight below the 10th percentile for gestational age. Poor start: Apgar score less than seven at 5 min after birth.	No, yes	Perined
Adverse experiences	Exposed to adverse experiences during the first 1000 days of life		
Loss of a parent	Death of a biological parent (2005–2008)	No, yes	SSD
Parental separation	Parental breakup during the first 1000 days of life (data from 2005 to 2008)	No, yes	SSD
Parental mental health illness	Based on mental healthcare services expenditures, categorised into lower or higher than population average (data from 2010)	No, yes	Vektis
Parental detention	Parental detention or parental registration as a crime suspect (data from 2005 to 2008)	No, yes	SSD

SSD, Social Statistical Datasets (Statistics Netherlands).

### Outcome: school performance

We investigated the association between children’s available resources in the first 1000 days of life and school performance at age 12. In short, in the Netherlands, after 8 years of primary education, children receive advice on the most suitable level of secondary education around age 12. Levels include prevocational secondary education, senior general secondary education (HAVO) or preuniversity education (VWO). The process follows a standard procedure: first, the child’s teacher provides a preliminary advice, based on his or her judgement of the child’s entire school career and skills (preliminary secondary school-level advice). Then, children undergo a formal academic achievement test, with a numeric outcome. These outcomes are mapped to the various secondary education levels (test-based secondary school-level advice).

1. If the test-based secondary school-level advice equals the preliminary secondary school-level advice, the preliminary secondary school-level advice becomes the final secondary school-level advice.
2. If the test-based secondary advice exceeds the preliminary secondary school-level advice, the teacher has the obligation to reconsider this preliminary secondary school-level advice together with parents to decide whether to adjust the preliminary secondary school-level advice to a higher final secondary school-level advice.
3. If the test-based secondary school-level advice is lower compared with the preliminary secondary school-level advice, the preliminary secondary school-level advice remains unchanged and becomes the final secondary school-level advice.

For this study, we used children’s final secondary school-level advice as a proxy for academic achievement, which takes all aspects of judgement into consideration (both the teacher’s judgement and formal testing). Recent evidence shows that children from high socioeconomic backgrounds receive higher preliminary secondary school-level advice than equally performing children from low socioeconomic backgrounds.^25^ Therefore, post hoc, we additionally associated children’s available resources with test-based secondary school-level advice to rule out this bias and to test the robustness of our conclusions.

Children who were born in 2006 were most likely to undergo academic achievement testing in 2017–2018. We also included data from the 2016–2017, 2018–2019 and 2019–2020 school years to include children who skipped or repeated 1 or 2 years.

### Statistical analysis

#### Latent class analysis

We used LCA to identify subgroups of available resources during the first 1000 days of life. LCA is a statistical data-driven approach that aims to identify unobserved (latent) clusters within a population by considering multiple variables concurrently.^26^ The idea is that a combination of relevant resources draws a more complete and in-depth picture of the first 1000 days of life and then focuses only on single types of resources. Therefore, LCA captures the multidimensionality of data on available resources in the first 1000 days of life. We estimated ten models for the data, from a one-class model to a ten-class model, with no prior assumption on the optimum number of classes. We decided on the optimum number of classes using multiple model fit statistics: (1) information criteria, such as the Bayesian information criterion (BIC), with a lower BIC implying a better model fit and (2) goodness-of-fit tests (L^2^ and bootstrapped L^2^), which indicate the amount of association among the variables that remain unexplained after estimating the model (the lower the value is, the better the fit of the model to the data). (3) (Bootstrapped) likelihood-ratio tests and (4) classification diagnostics, such as entropy, which indicate how accurately the model defines classes, with entropy values ranging from 0 to 1, with a value of one being ideal. We evaluated these fit statistics in conjunction with theoretical interpretability. Because of the large number of study participants, we acknowledged beforehand that statistical significance was less important and therefore evaluated the relative decrease in fit statistics. After we identified the best model, we assigned each child to a class (or cluster) of resources based on the maximum posterior probability. We described the classes using class proportions (the relative prevalence of each class) together with the item-response probabilities (the probability that an individual in a specific latent class will respond in a certain way to an observed item). In case of missing data, we performed complete-case analysis. We performed sensitivity analyses using two strategies. In strategy 1, we imputed missing data using multiple imputation. We created five imputed datasets using the package multiple imputation using chained equations and randomly chose one dataset for further analyses. The imputation model included all variables used in the analysis, as well as auxiliary variables related to missingness to improve imputations. We imputed binary variables using logistic regression and unordered categorical variables using polytomous logistic regression. In strategy 2, we used a population weight variable for educational attainment in our analyses, as constructed by Statistics Netherlands (strategy 2).

#### Association between latent class membership and school performance

We constructed three logistic regression models to calculate the OR and 95% CI of the association between the class of available resources during the first 1000 days of life and the highest level of secondary education (yes or no). We defined the highest level of secondary education as either HAVO advice or VWO advice, which prepare pupils for higher professional education and university studies. In model 1, we did not adjust for potential confounders. In model 2, we adjusted model 1 for parental education using the variable highest achieved parental education (low/intermediate/high). In model 3, we additionally adjusted model 2 for standardised disposable household income (low/moderate/high).

We considered a p<0.05 to indicate statistical significance. We used R-Studio, V.1.3.1056,^27^ for the statistical analyses and LatentGold, V.6.0.0 (Statistical Innovations, Belmont, USA), for the LCA. We reported our findings according to the Strengthening the Reporting of Observational Studies in Epidemiology (STROBE) Statement (see online supplemental table S1 for the STROBE checklist).^28^

## Results

### Study population

**Figure 1:**
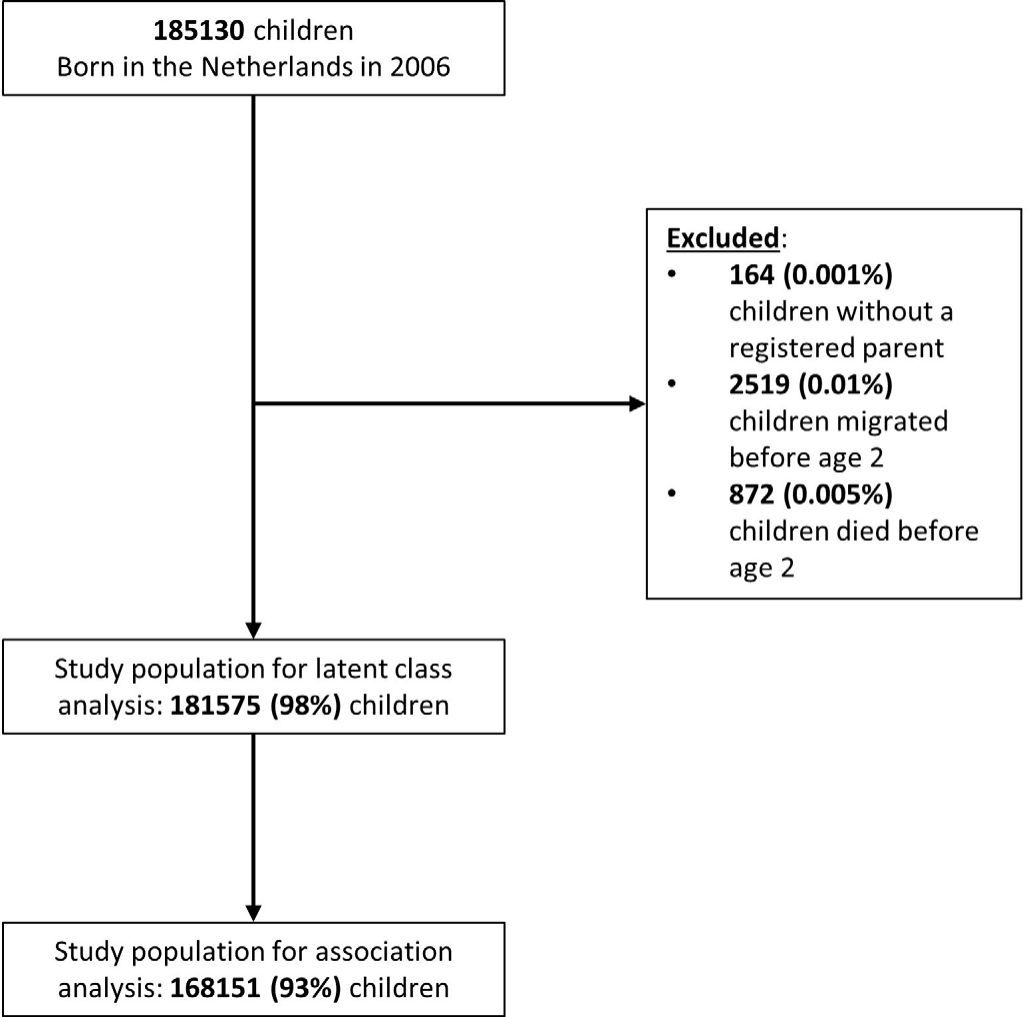
Cohort flow diagram.

Of the initial 185 130 children living in the Netherlands and born in 2006, we included 181 575 (98%) children (figure 1). We excluded 164 (0.001%) children who had no parents registered, 2519 (0.01%) children who migrated before their second birthday and 872 (0.01%) children who died before age 2.

The amount of missing data for each indicator variable was as follows (from high to low): parental educational level, 54 130 (30%); late initiation of antenatal care, 29 893 (17%) and household income, 1848 (1%). In total, 103 527 children (57%) had complete data on all variables and were included in LCA.

### Latent classes and labels

As expected, almost all fit statistics reached significance because of the large number of study participants. We considered the five-class model to be the best model based on the relative decrease in fit statistics (BIC), parsimony and model interpretability (online supplemental table S2 for fit statistics). For simplicity, we refer to the five classes as clusters 1, 2, 3, 4 and 5. Table 2 shows the class proportions and item-response probabilities for the five-class model of children’s available resources in the first 1000 days of life. We gave all clusters descriptive labels based on the degree of available resources during the first 1000 days of life.

**Table 2:** Class proportions and item-response probabilities for the five-class model of available resources in the first 1000 days of life

	Cluster 1 0.39	Cluster 2 0.27	Cluster 3 0.15	Cluster 4 0.15	Cluster 5 0.05	Population average
Label	Resource-richest	Resource-richest	Intermediate	Intermediate	Resource-poorest	
Socioeconomic characteristics						
Household income (standardised disposable)						
Low	0.19	0.00	0.98	0.10	0.79	0.28
Moderate	0.73	0.15	0.01	0.75	0.21	0.45
High	0.08	0.85	0.00	0.15	0.00	0.28
Parental highest achieved educational level						
Low	0.07	0.00	0.51	0.34	0.64	0.19
Intermediate	0.42	0.15	0.42	0.51	0.33	0.36
High	0.50	0.84	0.07	0.15	0.03	0.45
Pre- and perinatal biomedical factors						
Age of the mother (<21 years)						
No	1.00	1.00	0.97	0.96	0.81	0.98
Yes	0.00	0.00	0.03	0.04	0.19	0.02
Late antenatal care initiation						
No	0.94	0.91	0.82	0.84	0.71	0.89
Yes	0.06	0.09	0.18	0.16	0.29	0.11
Preterm birth or born small for gestational age, or poor start in life						
No	0.88	0.84	0.83	0.71	0.70	0.83
Yes	0.12	0.17	0.17	0.29	0.31	0.17
Adverse experiences						
Loss of a parent						
No	0.99	0.99	0.99	0.99	0.96	0.99
Yes	0.01	0.01	0.01	0.01	0.04	0.01
Parental separation						
No	0.98	0.98	0.94	0.95	0.85	0.97
Yes	0.02	0.02	0.06	0.05	0.15	0.03
Parental mental health illness						
No	0.93	0.95	0.86	0.88	0.65	0.91
Yes	0.07	0.05	0.14	0.12	0.35	0.09
Parental detention or registration as crime suspect						
No	0.99	0.99	0.95	0.95	0.66	0.97
Yes	0.01	0.01	0.05	0.05	0.34	0.03

Totals may not add up to one due to rounding.

Children in cluster 1 (39% of the population) and cluster 2 (27%) had lower-than population-average probabilities of prenatal and perinatal biomedical risk factors and lower-than population-average probabilities of adverse experiences during the first 1000 days of life. Among all the clusters, cluster 2 had the highest absolute probability of living with parents with high household income (0.85) and high education (0.84). Within cluster 1, children had the highest probability of living with parents with moderate household income (0.73) and high education (0.50). We categorised children in cluster 1 and cluster 2 as ‘resource-richest’.

Children in cluster 3 (15%) had the highest probability of living with parents with low household income (0.98) and low educational level (0.51), together with higher than population-average probabilities of prenatal and perinatal biomedical risk factors and adverse experiences in the first 1000 days of life. Children in cluster 4 (15%) grew up in an environment with intermediate household income (0.75), comparable to profile 1, but with intermediate parental education (0.51). Furthermore, children in cluster 4 had higher-than-population-average probabilities of prenatal and perinatal biomedical risk factors and greater-than-population-average chances of adverse experiences. We categorised both groups as having ‘intermediate resources’.

The children in cluster 5 (5%) mostly had a low household income (0.79), and parents were likely to have received a low level of education (0.64). Compared with children in all other clusters, children in cluster 5 had the highest probability of exposure to prenatal and perinatal biomedical factors (0.31 compared with 0.17 on average) and adverse experiences. For instance, children in this cluster were more than 10 times more likely to have a parent who had been detained (0.34 compared with 0.03 on average) and more than three times more likely to have a parent who suffered from mental health illnesses (0.35 compared with 0.09 on average). We labelled cluster 5 ‘resource-poorest’.

### Associations between available resources and school performance

For 168 151 children out of 181 575 children (93%), we had data on educational outcomes. Cluster 2 had the highest odds for the highest school performance. Compared with cluster 2, cluster 5, which included children with the fewest resources in the first 1000 days of life, had the lowest OR for the highest school performance (OR 0.13, 95% CI 0.11 to 0.14). After adjustment for parental education and household income, this association remained significant (adjusted OR 0.20, 95% CI 0.18 to 0.24) (table 3). Sensitivity analyses for missing data revealed no major differences between complete case analysis and sensitivity analyses using either multiple imputation for missing data, or weight variable for parental educational attainment (data not shown).

**Table 3:** ORs for highest secondary school level advice (HAVO/VWO) according to the available resources in the first 1000 days of life among Dutch children

	Model 1: no adjustments	Model 2: adjusted for parental education	Model 3: adjusted for parental education+family income
	OR (95% CI)	OR (95% CI)	OR (95% CI)
Cluster 1	0.52 (0.50 to 0.54)	0.61 (0.59 to 0.63)	0.69 (0.62 to 0.75)
Cluster 2	Reference group	Reference group	Reference group
Cluster 3	0.24 (0.23 to 0.25)	0.34 (0.33 to 0.36)	0.36 (0.34 to 0.39)
Cluster 4	0.23 (0.22 to 0.24)	0.32 (0.30 to 0.34)	0.35 (0.32 to 0.39)
Cluster 5	0.13 (0.11 to 0.14)	0.19 (0.17 to 0.22)	0.20 (0.18 to 0.24)

HAVO, senior general secondary education; VWO, preuniversity education.

In a post hoc sensitivity analysis, instead of using final secondary school-level advice, we associated available resources during the first 1000 days of life with test-based secondary school-level advice. We found similar but less pronounced results (online supplemental table S3). Compared with cluster 2, children in cluster 5, with the fewest resources in the first 1000 days of life, had the lowest OR for achieving test scores that corresponded to the highest secondary school-level advice (OR 0.15, 95% CI 0.13 to 0.17). Again, this was independent of parental education and household income (adjusted OR 0.49, 95% CI 0.40 to 0.60).

### Individual resource indicators and school performance

Most indicators that we included were independently associated with lower odds when present than when not present, except for a late start of antenatal care and a low Apgar score at 5 min after birth (table 4). Socioeconomic characteristics were the strongest predictor for receiving the highest academic achievement at the end of primary school.

**Table 4:** ORs for highest secondary school level advice (HAVO/VWO, yes or no) according to individual indicators in the first 1000 days of life among Dutch children

	OR (95% CI)
Socioeconomic characteristics	
Household income: low vs high	0.44 (0.43 to 0.46)
Household income: moderate vs high	0.54 (0.53 to 0.56)
Parental education: low vs high	0.23 (0.22 to 0.24)
Parental education: intermediate vs high	0.38 (0.37 to 0.39)
Pre- and perinatal biomedical factors	
Premature birth: yes vs no	0.85 (0.80 to 0.90)
Small for gestational age: yes vs no	0.74 (0.70 to 0.77)
Apgar <7 (5 min): yes vs no	0.86 (0.74 to 1.01)
Young age of the mother: yes vs no	0.67 (0.60 to 0.75)
Late start antenatal care: yes vs no	0.99 (0.95 to 1.04)
Adverse experiences	
Parental death: yes vs no	0.85 0.73 to 0.98)
Parental detention or suspect of crime: yes vs no	0.66 (0.60 to 0.72)
Parental separation: yes vs no	0.79 (0.73 to 0.86)
Parental mental illness: yes vs no	0.86 (0.82 to 0.90)

HAVO, senior general secondary education; VWO, preuniversity education.

## Discussion

This Dutch population-based birth cohort study aimed to investigate clustering of children’s resources in the first 1000 days of life and to what extent different risk factors/resources cluster and how such clustering affects later-life educational outcomes. We identified five clusters of resources during the first 1000 days of life, ranging from the resource-richest clusters (clusters 1 and 2, 66% of the study population) to the resource-poorest cluster (cluster 5, 5% of the population), with the latter having the highest probabilities of low socioeconomic resources, prenatal and perinatal biomedical risk factors and having experienced adverse childhood experiences in the first 1000 days. Compared with children in the resource-richest cluster, children with poor resource clustering across all three domains during the first 1000 days of life had markedly lower school performance at age 12, regardless of parental education and household income.

Clustering of poor resources across all three domains occurred in 5% of the population. In this cluster, labelled resource-poorest, 80% of the children grew up with low household income, and 64% of their parents had low educational attainment. These children had the highest probabilities of having prenatal and perinatal biomedical risks, as well as having experienced adverse childhood experiences in the first 1000 days of life. For example, 31% of the children in this cluster were born prematurely, born SGA or had a poor start in life (compared with 11% in the general population), 35% had parent(s) with a mental illness (compared with 9% in the general population) and 34% of the children had a detained parent or a parent who was registered as a crime suspect (compared with 3% in the general population). Although the absolute probability for loss of a parent in this cluster was low (4%), this was four times greater than that of the general population (1%).

The findings of the current study are in line with those of previous clustering studies, showing that clustering of poor resources across different domains is related to poorer developmental and educational outcomes. For example, a population-wide Tasmanian cohort study (5440 children) revealed that children with clustering of risk factors at birth in all domains (birth, sociodemographic and health behaviour risks) had the highest probability of developmental vulnerability at age 5 compared with children in the low-risk reference group (OR 3.29, 95% CI 2.10 to 5.16).^20^ In contrast to our study, researchers used a nationwide available measure of child development at age 5, including the domains of physical health and well-being, social competence, emotional maturity, language and cognitive skills, communication skills and general knowledge, and defined developmental vulnerability as a score below the 10th percentile. Although Dutch preventive child and youth healthcare services routinely collect these types of developmental data, these data are unavailable in Dutch registries and therefore could not be included in our analyses.

A cohort study from Iceland (1151 children) included risk factors beyond birth, including maternal smoking during pregnancy, parental disability status, birth to a young mother, number of children in the household, family income, number of visits to school nurses and reports of maltreatment, and highlighted the cumulative impact of multiple risk factors on academic achievement (test scores in Icelandic language and mathematics in fourth and seventh grades). The more early-life risk factors a child accumulated over the early life course, the poorer his or her academic outcomes were.^19^ Although the above-described studies included different risk factors in early life, they show consistent evidence for an association between early-life adversity and later-life development and educational outcomes.

We used final secondary school-level advice as a proxy for school performance. There is also evidence from the Dutch schooling system that suggests that schoolteachers provide higher secondary school-level advice for children from high socioeconomic backgrounds than for equally performing children from low socioeconomic backgrounds.^25^ To study this potential bias, we additionally investigated the association between the identified resource clusters and secondary school-level advice based only on formal testing. In contrast to the final secondary school-level advice, this advice is based only on formal testing and does not include the potential biased judgement of the teacher. On the other hand, it is more subject to time-specific conditions. Our conclusions did not change, as children who grew up in the resource-poorest cluster (cluster 5) had lower odds of receiving the highest level of secondary school advice based on the test (OR 0.15, 95% CI 0.13 to 0.17) than did children in the resource-richest cluster (cluster 2). This effect remained after adjustment for parental education and household income (adjusted OR 0.49, 95% CI 0.40 to 0.60), although the differences were less marked.

The current study provides evidence that resources within the first 1000 days of life are associated with later-life educational outcomes. Children who grew up in the resource-poorest environment (cluster 5) had an 80% reduction in school performance compared with children who grew up in the resource-richest environment (cluster 2), regardless of socioeconomic resources, such as parental education or household income. This suggests that these children, in the long termv, may not be able to achieve their full developmental potential and may attain lower levels of education. This may have long-term implications, as previous evidence has demonstrated that expected achievement serves as a relevant predictor of future academic achievement.^29 30^ Higher educational attainment is strongly associated with life expectancy and health, as people with a lower level of education have higher mortality and poorer health than people with a higher level of education.^31 32^ Moreover, educational attainment plays an important role in health, as education is the main element of human capital by shaping opportunities, employment and income.^33 34^

### Individual factors

In addition to LCA, we performed multivariate analyses including individual resource indicators in the first 1000 days of life and investigated the associations with school performance. Consistent with previous studies, the present study revealed that socioeconomic characteristics were the strongest indicators of school performance, with low parental education being the strongest individual predictor.^35^ Moreover, children born preterm or born SGA, as well as children born to young mothers, had poorer school performance than children who were born full term or with normal birth weight, consistent with existing evidence.^10 11 13^ The current study also revealed this association for children born to young mothers (younger than 21 years old at the time of birth), which is in line with previous studies. The current study found evidence that all individual adverse childhood experiences included in this analysis were independently associated with poorer school performance, with parental detention or being a crime suspect showing the strongest association with poorer school performance. Surprisingly, previous evidence on the impact of parental incarceration on children’s academic outcomes has been inconsistent.^36 37^ Researchers from the Pittsburgh Youth Study suggested that the lack of effects of parental incarceration on poor academic performance may be explained by the small consequences in the context of other risk experiences. Very few studies have examined changes in academic performance before and after parental incarceration.

The findings of the current study suggest that socioeconomic resources in the first 1000 days of life only partially explain later-life educational outcomes. It appears that the multidimensionality of resources, including pregnancy and birth-related adversities, as well as adverse childhood experiences in the first 1000 days of life, together associate with poorer educational outcomes. For example, children in clusters 3 and 5 had low household income and low parental education. In contrast to cluster 3, cluster 5 had the highest probabilities of pregnancy-related and birth-related adversity and adverse childhood experiences, which were significantly associated with lower educational performance (OR 0.20 vs 0.36). If we find similar associations with health-related outcomes, this may imply that policies aimed at developing human potential should focus on strengthening resources across multiple domains. Yet, a recent intervention study that evaluated the effect of multidomain interventions from preconception through early childhood found modest improvements in child neurodevelopment at age 24 months.^38^

### Strengths and limitations

A major strength of this study is that we included an entire birth cohort of over 180 000 children (and their parents) in the Netherlands to investigate the most crucial developmental phase. Due to this study design, using large representative register-based data, we believe that the overall generalisability of our results is high. In comparison to scientific research in general, we know that not all individuals participate equally, thereby generating selection bias.^39^ However, we acknowledge that we missed specific vulnerable groups of children, such as refugee children and children of undocumented immigrants. Consequently, we cannot draw any conclusions on these groups. Another strength is that we drew a multidimensional picture of the first 1000 days of life, as we focused on multiple relevant resources across domains. We included nine indicators based on a review of the literature and theoretical considerations and by using predefined guiding principles.

We also have to acknowledge some limitations. One of the limitations of using registry data is that we were restricted to data available in Dutch registries. Therefore, we were unable to capture all aspects of the environment in which children develop during their first 1000 days. Exposures to maternal malnutrition, noise, air pollution, toxins and pesticides—which are all known to affect development—were not included in our analyses due to unavailability.^40^ For example, prenatal alcohol exposure has been associated with poorer academic performance, which is suggested to be caused by atypical brain development.^41 42^ Because severe prenatal alcohol exposure is associated with low socioeconomic status, which we included in this study, we believe the impact of this omission is limited.^43^ Moreover, we were unable to investigate children’s developmental trajectories as an outcome, as these data are unavailable in Dutch registries. Moreover, we lacked data on parental mental healthcare expenditures during the first 1000 days of life, and instead, we relied on data available from 2010 as a proxy. This may have resulted in missed mental healthcare costs during the first 1000 days of life, which returned to normal in 2010. We believe that this is less of a limitation, as mental healthcare costs in 2010 will be associated with mental healthcare costs in offspring’s first 1000 days of life. Last, not all adverse childhood experiences (such as domestic violence) are registered, which may have affected our estimates. Second, we had a fair amount of missing data, in particular for parental educational attainment, which may have introduced selection bias using complete cases analysis. However, sensitivity analyses using the population weight variable for educational attainment and using multiple imputation did not alter our conclusions and therefore appear to be less of a limitation. Third, we used LCA for exploratory analysis to understand the clustering of resources within the first 1000 days of life. We acknowledge the shortcomings of this model, as classification errors were present (27%). We did not intend to use these clusters to categorise individuals but rather to study how available resources cluster and, in the future, to explore which clusters of children may benefit most from early-life public health policies. Fourth, we used secondary school-level advice as a proxy for academic achievement. We acknowledge that advice for secondary education does not imply that children actually follow and complete that particular trajectory. A future study including data on the attained educational level in this cohort would be useful to validate this. Finally, the current study aimed to investigate the relationship between resources within the first 1000 days of life and later-life educational outcomes. However, it remains a methodological challenge to disentangle the impact of socioeconomic resources within the first 1000 days of life from the impact of socioeconomic resources beyond this period. However, existing research on brain development recognises the first 1000 days of life as a critical and sensitive period for brain development.^44^ Therefore, we believe that the impact of socioeconomic variables beyond age 2 on educational performance is relatively low.

### Future recommendations

In future studies, we will focus on the association between available resources within the first 1000 days of life and other relevant outcomes, such as children’s developmental trajectories and health-related outcomes. This study illustrates how existing data routinely collected in the Netherlands can be used in research on resources within the first 1000 days of life and later-life educational outcomes. We firmly advocate for prioritising the creation of a national longitudinal dataset on child development in the Netherlands, as the Dutch preventive child and youth healthcare service routinely collects these data.^45^ Population-based longitudinal data on child development will give public health researchers the opportunity to study the relationship between available resources in the first 1000 days of life and subsequent child development at the population level. Moreover, it will provide policy-makers with the opportunity to monitor and evaluate interventions. According to the Child and Youth Act, it is the responsibility of Dutch municipalities to provide preventive child and youth healthcare in the Netherlands. Therefore, to obtain a national longitudinal dataset, all Dutch municipalities should agree to submit their data to a national registry. While this study focuses on the Netherlands, the relationship between early-life circumstances and educational outcomes is relevant across various contexts. However, the impact of early-life factors on education may vary depending on national policies, healthcare systems and social safety nets.

## Conclusions

In conclusion, in this large population-based registry study, clustering or poor resources across different domains during the first 1000 days of life were associated with poorer school performance at age 12, independent of the education level of parents and household income. This suggests that children who grow up in a resource-poor environment in the first 1000 days of life do not achieve their full developmental and educational potential. If we find similar associations with health-related outcomes, this may imply that policies aimed at developing human potential should focus on strengthening resources across multiple domains.

## Supplementary material

10.1136/bmjph-2024-002176 online supplemental file 1

## Data Availability

Data may be obtained from a third party and are not publicly available.
