# Supplementary material for: Clustering of circumstances during the first 1000 days after conception and their association with school performance: a population-based cohort study from the Netherlands
Source: BMJ Public Health. 2025 Aug 17;3(2):e002176. doi: 10.1136/bmjph-2024-002176 (PMC12359527; doi:10.1136/bmjph-2024-002176)
Supplement: online supplemental file 1 [file bmjph-3-2-s001.docx]

**Supplementary data**

**Table S1:** **STROBE checklist**

| **Section/Topic** | Item # | Recommendation | Reported on page # |
| --- | --- | --- | --- |
| **Title and abstract** | 1 | (*a*) Indicate the study’s design with a commonly used term in the title or the abstract | 1 |
|  |  | (*b*) Provide in the abstract an informative and balanced summary of what was done and what was found | 1 |
| Introduction | | |  |
| Background/rationale | 2 | Explain the scientific background and rationale for the investigation being reported | 3 |
| Objectives | 3 | State specific objectives, including any prespecified hypotheses | 3 |
| Methods | | |  |
| Study design | 4 | Present key elements of study design early in the paper | 3 |
| Setting | 5 | Describe the setting, locations, and relevant dates, including periods of recruitment, exposure, follow-up, and data collection | 3, 4 |
| Participants | 6 | (*a*) Give the eligibility criteria, and the sources and methods of selection of participants. Describe methods of follow-up | 4 |
|  |  | (*b*) For matched studies, give matching criteria and number of exposed and unexposed | NA |
| Variables | 7 | Clearly define all outcomes, exposures, predictors, potential confounders, and effect modifiers. Give diagnostic criteria, if applicable | 4-6 |
| Data sources/ measurement | 8* | For each variable of interest, give sources of data and details of methods of assessment (measurement). Describe comparability of assessment methods if there is more than one group | 5 |
| Bias | 9 | Describe any efforts to address potential sources of bias | 6, 7 |
| Study size | 10 | Explain how the study size was arrived at | 7, 8 |
| Quantitative variables | 11 | Explain how quantitative variables were handled in the analyses. If applicable, describe which groupings were chosen and why | 5 |
| Statistical methods | 12 | (*a*) Describe all statistical methods, including those used to control for confounding | 6, 7 |
|  |  | (*b*) Describe any methods used to examine subgroups and interactions | NA |
|  |  | (*c*) Explain how missing data were addressed | 7 |
|  |  | (*d*) If applicable, explain how loss to follow-up was addressed | NA |
|  |  | (*e*) Describe any sensitivity analyses | 6 |
| Results | | |  |
| Participants | 13* | (a) Report numbers of individuals at each stage of study—eg numbers potentially eligible, examined for eligibility, confirmed eligible, included in the study, completing follow-up, and analysed | 7, 8 |
|  |  | (b) Give reasons for non-participation at each stage | 8 |
|  |  | (c) Consider use of a flow diagram | 8 |
| Descriptive data | 14* | (a) Give characteristics of study participants (eg demographic, clinical, social) and information on exposures and potential confounders | 7 |
|  |  | (b) Indicate number of participants with missing data for each variable of interest | 7 |
|  |  | (c) Summarise follow-up time (eg, average and total amount) | NA |
| Outcome data | 15* | Report numbers of outcome events or summary measures over time | NA |
| Main results | 16 | (*a*) Give unadjusted estimates and, if applicable, confounder-adjusted estimates and their precision (eg, 95% confidence interval). Make clear which confounders were adjusted for and why they were included | 10, 11 |
|  |  | (*b*) Report category boundaries when continuous variables were categorized | NA |
|  |  | (*c*) If relevant, consider translating estimates of relative risk into absolute risk for a meaningful time period | NA |
| Other analyses | 17 | Report other analyses done—eg analyses of subgroups and interactions, and sensitivity analyses | S2 |
| Discussion |  |  |  |
| Key results | 18 | Summarise key results with reference to study objectives | 12 |
| **Limitations** |  |  |  |
| Interpretation | 20 | Give a cautious overall interpretation of results considering objectives, limitations, multiplicity of analyses, results from similar studies, and other relevant evidence | 12-15 |
| Generalisability | 21 | Discuss the generalisability (external validity) of the study results | 13 |
| Other information |  |  |  |
| Funding | 22 | Give the source of funding and the role of the funders for the present study and, if applicable, for the original study on which the present article is based | 15 |

**Table S2:** Model fit statistics for identifying the latent class model

|  | **log-likelihood value (LL)** | **Bayesian Information Criterion (BIC)** | **Relative decrease BIC** | **Akaike Information Criterion (AIC)** | **Number of parameters** | **L²** | **p-value** | **Bootstrap p-value** | **Max. Bivariate residuals** | **Vuong-Lo-Mendell-Rubin** | **Vuong-Lo-Mendell-Rubin**  **p-value** | **Likelihood ratio test** | **Bootstrap p-value** | **Classification error** | **Entropy R²** |
| --- | --- | --- | --- | --- | --- | --- | --- | --- | --- | --- | --- | --- | --- | --- | --- |
| **1-Cluster** | -356,382 | 712,891 |  | 712,786 | 11 | 34862 | 0.000 | 0.000 | 21333 |  |  |  |  | 0.00 | 1.00 |
| **2-Cluster** | -341,597 | 683,435 | 29456 | 683,236 | 21 | 5292 | 0.000 | 0.000 | 443 | 29570 | 0.000 | 29570 | 0.000 | 0.116 | 0.606 |
| **3-Cluster** | -340,035 | 680,425 | 3010 | 680,131 | 31 | 2167 | 0.000 | 0.000 | 164 | 3125 | 0.000 | 3125 | 0.000 | 0.243 | 0.492 |
| **4-Cluster** | -339,802 | 680,075 | 350 | 679,686 | 41 | 1702 | 0.000 | 0.000 | 111 | 465 | 0.000 | 465 | 0.000 | 0.240 | 0.500 |
| **5-Cluster** | -339,626 | 679,838 | 237 | 679,355 | 51 | 1350 | 0.000 | 0.000 | 55 | 352 | 0.000 | 352 | 0.000 | 0.272 | 0.510 |
| **6-Cluster** | -339,555 | 679,811 | 27 | 679,232 | 61 | 1208 | 0.0071 | 0.000 | 42 | 142 | 0.000 | 142 | 0.000 | 0.313 | 0.460 |
| **7-Cluster** | -339,481 | 679,776 | 35 | 679,103 | 71 | 1059 | 0.68 | 0.000 | 11 | 149 | 40.531 | 149 | 0.000 | 0.389 | 0.411 |
| **8-Cluster** | -339,442 | 679,813 | +37 | 679,045 | 81 | 981 | 0.98 | 0.000 | 10 | 78 | 0.000 | 78 | 0.000 | 0.453 | 0.379 |
| **9-Cluster** | -339,398 | 679,841 | +28 | 678,979 | 91 | 894 | 1.00 | 0.000 | 11 | 86 | 0.000 | 86 | 0.000 | 0.387 | 0.469 |
| **10-Cluster** | -339,380 | 679,919 | +78 | 678,962 | 101 | 857 | 1.00 | 0.000 | 6 | 37 | 2.232 | 37 | 0.000 | 0.409 | 0.438 |

**Table S3:** Odds ratios for highest secondary school level advice (HAVO/VWO, yes or no) based on test results, according to the available resources in the first 1000 days of life among Dutch children.

|  | **Model 1:**  **No adjustments** | **Model 2:**  **Adjusted for parental education** | **Model 3:**  **Adjusted for parental education + family income** |
| --- | --- | --- | --- |
|  | **OR (95% CI)** | **OR (95% CI)** | **OR (95% CI)** |
|  |  |  |  |
| **Cluster 1** | 0.55 (0.53 – 0.56) | 0.62 (0.60 – 0.64) | 0.96 (0.83-1.10) |
| **Cluster 2** | Reference group | Reference group | Reference group |
| **Cluster 3** | 0.25 (0.24 – 0.26) | 0.48 (0.45 – 0.51) | 0.82 (0.69- 0.96) |
| **Cluster 4** | 0.25 (0.24 – 0.27) | 0.53 (0.49 – 0.57) | 0.76 (0.66 – 0.87) |
| **Cluster 5** | 0.15 (0.13 – 0.17) | 0.29 (0.25 – 0.33) | 0.49 (0.40 – 0.60) |
